# Identification of a PORCN c.1093C>T (p.Arg365Trp) Variant in a 12‐Year‐Old Girl With Goltz–Gorlin Syndrome

**DOI:** 10.1002/ccr3.71592

**Published:** 2026-02-02

**Authors:** Anna Bolzon, Francesca Caroppo, Lisa Passaglia, Francesca Boaretto, Leonardo Salviati, Anna Belloni Fortina

**Affiliations:** ^1^ Dermatology Unit, Department of Medicine (DIMED) University of Padua Padua Italy; ^2^ Pediatric Dermatology Regional Center, Department of Women and Children's Health University of Padua Padua Italy; ^3^ European Network for Rare Skin Disorders (ERN‐Skin) Padua Italy; ^4^ Clinical Genetics Unit, Department of Women's and Children's Health University of Padova Padova Italy; ^5^ Institute of Pediatric Research Città Della Speranza Padua Italy

**Keywords:** genetic diseases, Goltz–Gorlin syndrome, mutation, PORCN, rare skin diseases, Wnt signaling pathway

## Abstract

We report the first female case of Goltz–Gorlin syndrome with the PORCN c.1093C>T (p.Arg365Trp) variant, previously described only in a male with Klinefelter syndrome. This case expands the known phenotypic and genotypic spectrum of FDH.

**Abbreviations**
FDH: focal dermal hypoplasia
PORCN: porcupine O‐acyltransferase

## 1 Introduction

Goltz–Gorlin syndrome, also known as focal dermal hypoplasia (FDH), is a rare ectodermal dysplasia syndrome primarily affecting the skin, skeleton, and eyes [1]. It is an X‐linked dominant disorder caused by variants in the *PORCN* (porcupine O‐acyltransferase) gene. This gene encodes the porcupine O‐acyltransferase, a protein involved in the Wnt/β‐catenin signaling pathway, which is important for embryonic development [1].

Over 90% of affected patients are female, as *PORCN* pathogenic variants are typically lethal before birth in males with non‐mosaic hemizygous mutations. Rare male cases are generally attributed either to somatic mosaicism arising from postzygotic variants or to the presence of Klinefelter syndrome [2]. Furthermore, males carrying hypomorphic *PORCN* variants have also been reported [3].

*PORCN*‐related disorders show variable phenotypic severity in females, which can be influenced by mechanisms such as skewed X‐chromosome inactivation or somatic mosaicism [4].

Despite approximately 300 cases reported in the literature to date, the true prevalence remains largely unknown [1].

Clinical manifestations of Goltz–Gorlin syndrome are variable among patients, depending on the proportion and distribution of cells expressing the mutant X chromosome. Skin lesions follow Blaschko's lines and include streaks of vermiculate dermal atrophy, with variable subcutaneous fat herniation, telangiectasias, erosions, and hypo‐ or hyperpigmentation. Other ectodermal findings may include raspberry‐like papillomas more often on the lips and anogenital regions but also in the pharynx and respiratory mucosa, dysplastic nails, brittle and sparse hair, limb malformations including ectrodactyly, oligodactyly, polydactyly, and syndactyly, oral and dental abnormalities such as cleft palate and hypodontia, ocular defects such as colobomas, cataracts, microphthalmia or anophthalmia, and dysmorphic facies (asymmetry, thin upper lip, and malformed ears). Central nervous system and urogenital anomalies are reported [5]. No clear genotype–phenotype correlation is described [6].

We present the case of a 12‐year‐old female with a rare PORCN mutation previously reported only in a male patient with karyotype 47,XXY affected by Klinefelter syndrome, who exhibited the classic features of FDH, contributing to the expanding understanding of this rare syndrome.

## 2 Case History/Examination

**FIGURE 1:**
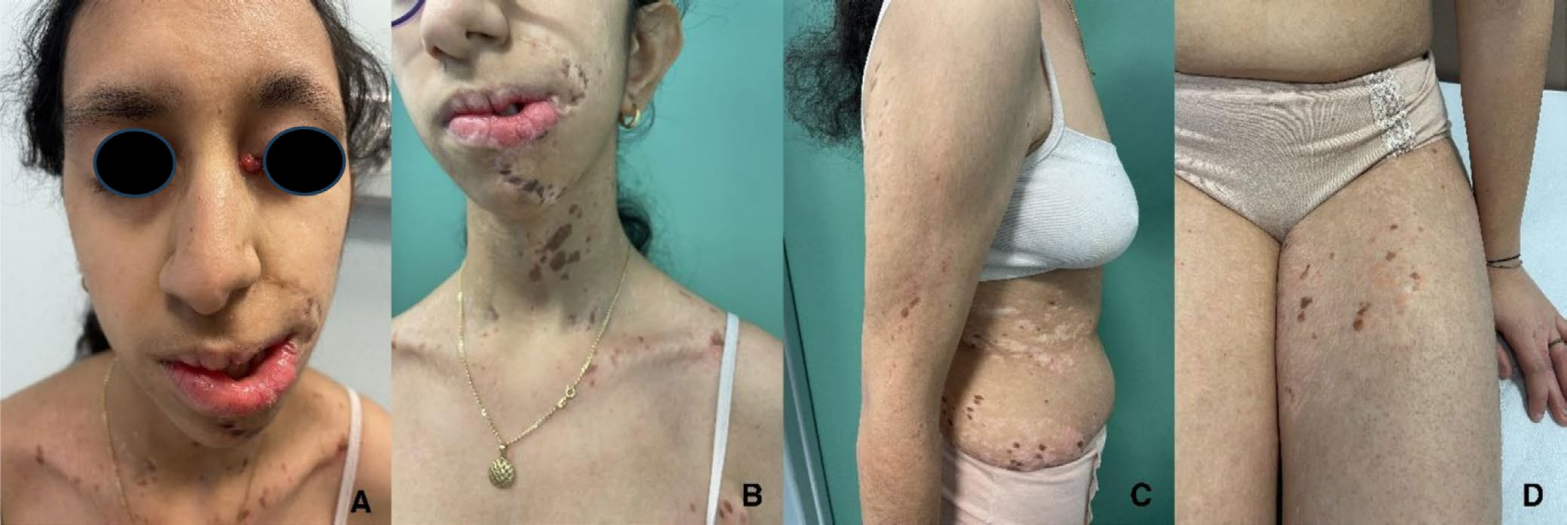
(A) Facial asymmetry, erythematous raspberry‐like papilloma on the left superior eyelid. (B–D) Vermiculated hypopigmented atrophic patches with focal subcutaneous fat protrusion and hyperpigmented areas on the face, neck, trunk, and extremities.

**FIGURE 2:**
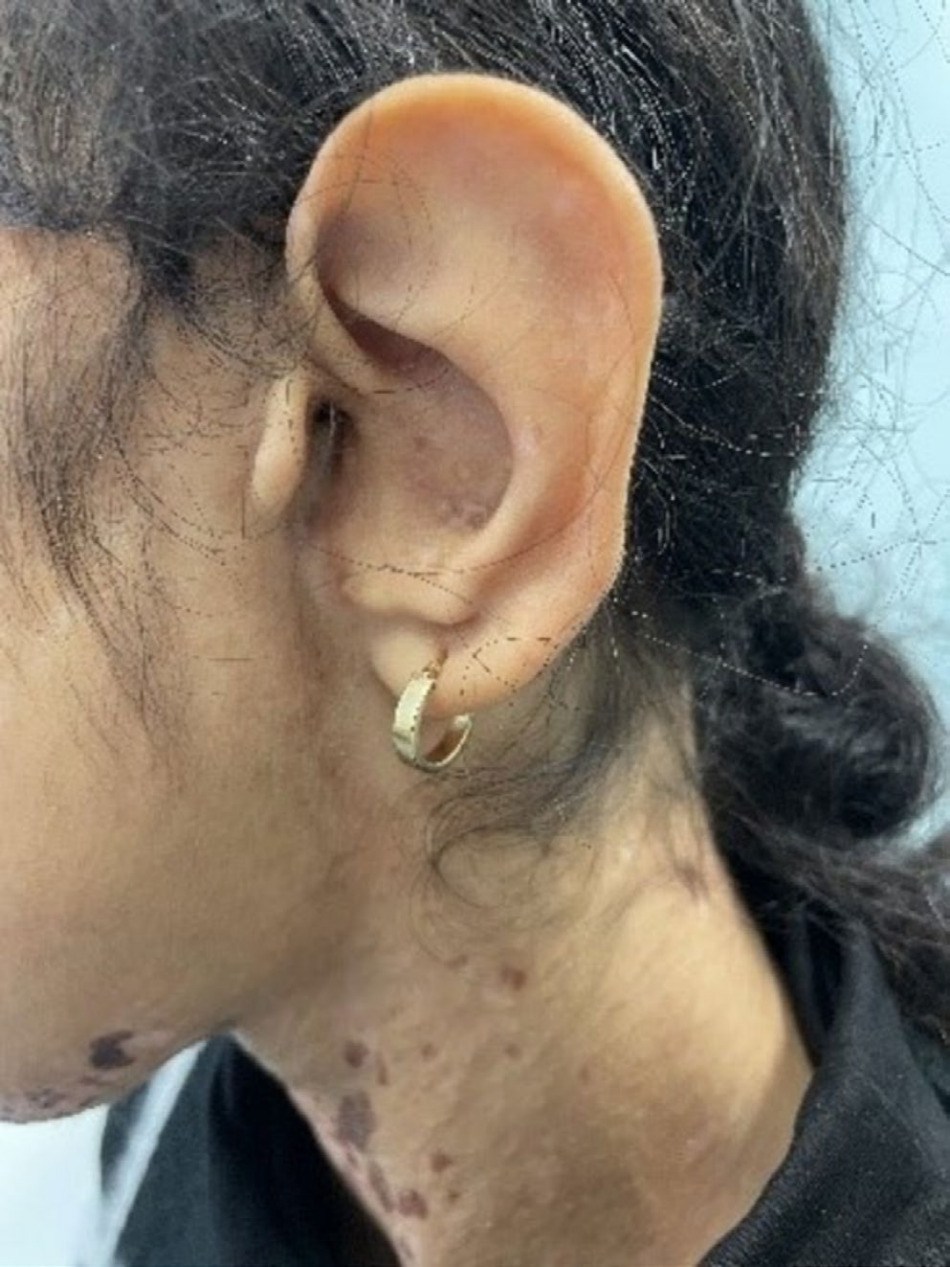
Left earlobe malformation.

**FIGURE 3:**
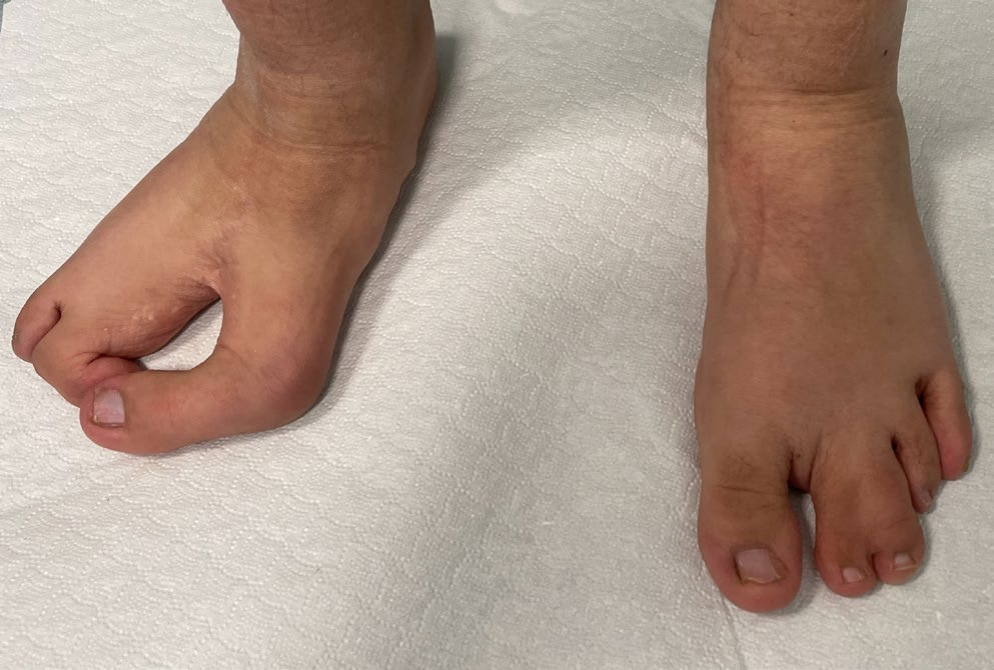
Feet ectrodactyly (also called cleft feet) and syndactyly.

**FIGURE 4:**
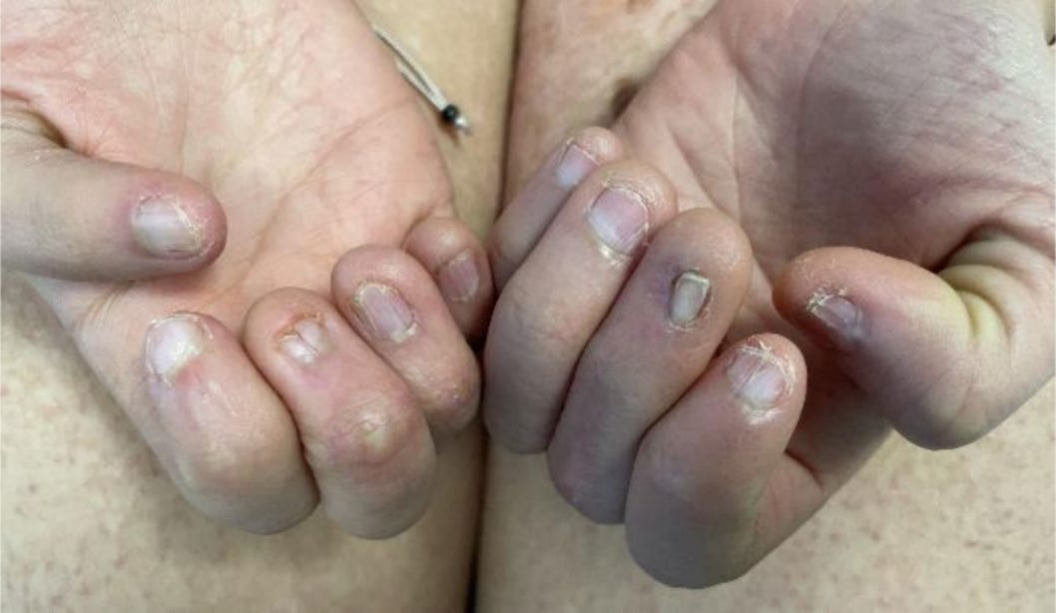
Longitudinal ridging of the nail plate and micronichia of the nails of both hands.

A 12‐year‐old female patient was referred to the Department of Pediatric Dermatology at the University of Padua. She presented with facial asymmetry, hypopigmented atrophic patches arranged in a linear distribution on the left side of her face and neck, and following Blaschko's lines on her trunk and arms. Focal subcutaneous fat protrusion presenting as soft yellow nodules was observed, along with telangiectasias and areas of hyperpigmentation. Additionally, she exhibited erythematous raspberry‐like papillomas on her upper left eyelid and pharynx. Eye involvement with coloboma and dental agenesis has been diagnosed. Limb abnormalities were present, with ectrodactyly and syndactyly of the feet. Nail involvement was observed, with longitudinal ridging of the nail plate and micronychia of the nails of both hands (Figures 1, 2, 3, 4).

**FIGURE 5:**
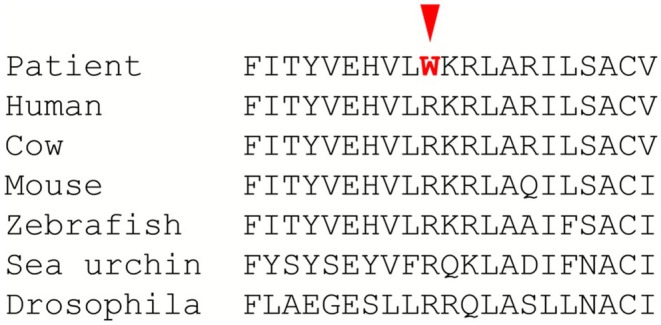
Alignment of PORCN proteins from different eukaryotic species. Arginine 365 is conserved in virtually all organisms with a PORCN ortholog.

## 3 Differential Diagnosis

The distinctive cutaneous findings of Goltz–Gorlin syndrome, especially linear dermal atrophy and fat herniations, help clinicians distinguish it from other genodermatoses.

Among these, MIDAS syndrome (microphthalmia, dermal aplasia, and sclerocornea), also referred to as MLS syndrome (microphthalmia with linear skin defects), is notable for linear skin atrophy restricted to the face and neck, in contrast to focal dermal hypoplasia, which also affects distal extremities [5].

Proteus syndrome is another condition to be considered in the differential diagnosis. It may present with patchy dermal atrophy and prominent subcutaneous veins. Still, it also exhibits additional cutaneous manifestations distinct from focal dermal hypoplasia, including epidermal nevi, cerebriform connective tissue nevi on the palms or soles, slow‐flow vascular malformations (particularly port‐wine stains), and abnormal fat distribution such as lipomas and areas of lipoatrophy [5].

## 4 Investigations

Genetic testing identified a de novo PORCN heterozygous variant, NM_203475.1:c.1093C>T in exon 13, leading to the amino acid substitution (p.Arg365Trp) in the PORCN protein.

Sequencing was carried out using an Illumina NextSeq 550 sequencer. Libraries were prepared using the Illumina TruSight ONE Expanded kit according to the standard protocol.

This variant is absent from version 4.1 of the Genome Aggregation Database (gnomAD). It has previously been reported in a male patient with Klinefelter syndrome (47,XXY karyotype) and clinical manifestations suggestive of FDH [2]. Different substitutions affecting this residue (p.Arg365Gln and p.Arg365Gly) have been identified in individuals diagnosed with PORCN‐related conditions.

The variant can be classified as pathogenic (ACMG class 5) based on the following criteria: PS2 (maternity and paternity were confirmed), PM2 (the variant is absent in the population database gnomAD v4.1 MAF = 0), PM5 (at least two other missense substitutions affecting this codon have been reported as pathogenic), PP3 (the Franklin meta‐prediction tool [https://franklin.genoox.com] suggests a deleterious role for the variant with a score of 0.842) and PM1 (different substitutions affecting this residue have been identified in individuals diagnosed with PORCN‐related conditions). Furthermore, the residue is highly conserved during evolution, indicating that it is critical for PORCN function and that any change at this codon affects protein function (Figure 5).

## 5 Therapy

Currently, no specific therapy is available for FDH. Management is primarily supportive and aimed at optimizing quality of life, with appropriate referrals to subspecialists based on the patient's associated anomalies [5].

In our case, the patient is under multidisciplinary follow‐up. Orthopedic corrective procedures have been performed for cleft foot deformity, while potential spinal anomalies are being monitored. Additionally, she is under ophthalmologic follow‐up for coloboma and raspberry‐like eyelid papillomas, with routine evaluations to monitor visual function and assess the need for surgical excision of papillomas.

## 6 Discussion

FDH is a rare genodermatosis related to variants affecting the gene encoding the protein PORCN. The *PORCN* gene is located on the short arm of the X chromosome (Xp11.23). It encodes a 461‐amino acid 52‐kDa endoplasmic reticulum protein, the porcupine O protein, which is important for the modification and secretion of Wnt proteins [2, 7]. Wnt proteins are critical for interactions between ectoderm and mesoderm during embryogenesis [8]. However, further research is required to elucidate the molecular mechanisms by which PORCN inactivation affects Wnt signaling and the manifestations of FDH [9].

The vast majority of affected individuals are female (approximately 90%), who are either heterozygous or, more rarely, mosaic for pathogenic variants in the *PORCN* gene. Typically, *PORCN*‐related developmental disorders show high penetrance in females; however, phenotypic severity can be attenuated in some cases due to skewed X‐chromosome inactivation or the presence of hypomorphic *PORCN* variants. Existing literature suggests that the degree of X‐chromosome inactivation correlates with phenotype severity in certain familial cases [8].

In males, the condition is prenatally lethal in those with constitutional hemizygous variants. Nevertheless, approximately 10% of live‐born affected males carry mosaic postzygotic pathogenic variants of the *PORCN* gene or are affected by Klinefelter syndrome [2]. Recently, males harboring hypomorphic *PORCN* variants have been reported [3].

To date, the Leiden Open Variation Database (LOVD) lists 282 distinct PORCN variants (https://databases.lovd.nl/shared/genes/PORCN, accessed January 8, 2025), including five cases of the c.1093C>T variant. Of these, only one has been reported in the literature, namely a male affected by Klinefelter syndrome (47,XXY karyotype).

We report a case of focal dermal hypoplasia in a 12‐year‐old girl with a de novo heterozygous single nucleotide substitution (C to T) at nucleotide 1093 of PORCN. This variant has never been described in female patients with focal dermal hypoplasia.

In conclusion, the identification of the c.1093C>T variant in the *PORCN* gene in a female patient expands the known genetic and phenotypic spectrum of FDH. The identification of any new mutations, as in our patient, also contributes to the expansion of the PORCN mutation database in FDH.

## Author Contributions

**Anna Bolzon:** conceptualization, data curation, methodology, writing – original draft, writing – review and editing. **Francesca Caroppo:** conceptualization, investigation, methodology, supervision, validation, writing – review and editing. **Lisa Passaglia:** funding acquisition, investigation, writing – original draft. **Leonardo Salviati:** formal analysis, funding acquisition, investigation, methodology, supervision, validation. **Anna Belloni Fortina:** investigation, methodology, supervision, validation. **Francesca Boaretto:** funding acquisition, investigation, methodology, writing – review and editing.

## Funding

The authors have nothing to report.

## Disclosure

No AI and AI‐assisted technologies have been used to write this work.

## Consent

The authors obtained written consent from the patient for their photographs and medical information to be published in print and online, and with the understanding that this information may be publicly available. Patient consent forms were not provided to the journal but are retained by the authors.

## Conflicts of Interest

The authors declare no conflicts of interest.

## Data Availability

Data sharing is applicable to this work.
